# Circular RNA *cIARS* regulates ferroptosis in HCC cells through interacting with RNA binding protein ALKBH5

**DOI:** 10.1038/s41420-020-00306-x

**Published:** 2020-08-07

**Authors:** Zhiqian Liu, Qi Wang, Xin Wang, Zongzhen Xu, Xiaoqing Wei, Jie Li

**Affiliations:** 1 Department of Hepatobiliary Surgery, The First Affiliated Hospital of Shandong First Medical University, 250014 Jinan, Shandong China; 2 Department of Hepatobiliary Surgery, Shandong Provincial Qianfoshan Hospital, Shandong University, 250014 Jinan, Shandong China; 3 Department of Reproduction Medicine, Jinan Maternal and Child Health Care Hospital Affiliated to Shandong First Medical University, 250001 Jinan, Shandong China

**Keywords:** Targeted therapies, Cell death, Autophagy, Targeted therapies, Cell death

## Abstract

Circular RNAs (circRNAs) are a novel and unique class of noncoding RNAs that are back-spliced from pre-mRNAs. It has been confirmed that circRNAs are involved in various malignant behaviors of hepatocellular carcinoma (HCC). However, the role of circRNA in the regulation of ferroptosis and the underlying mechanism remain unknown. Here, *cIARS* (hsa_circ_0008367) was found to be the most highly expressed circRNA after sorafenib (SF) treatment in HCC cells. Small interfering RNA against *cIARS* (si-*cIARS*) significantly suppressed the cellular sensitivity to SF or Erastin through inactivating ferroptosis, which may be partially attributed to the inhibition of autophagy and ferritinophagy. Prediction analysis and mechanistic identification revealed that *cIARS* physically interacted with RNA binding protein (RBP) ALKBH5, which was a negative regulator of autophagic flux in HCC. The dissociation of BCL-2/BECN1 complex, mediated by ALKBH5 silencing was effectively blocked by si-*cIARS*. Furthermore, the inhibition of ferroptotic events, autophagic flux and ferritinophagy resulted from si-*cIARS*, were significantly rescued by ALKBH5 downregulation. Overall, *cIARS* may be an important circRNA, positively regulating SF-induced ferroptosis through suppressing the ALKBH5-mediated autophagy inhibition.

## Introduction

HCC is a highly refractory and prevalent cancer worldwide, with ~841,000 new cases and 782,000 deaths every year^1^. Rapid advances in diagnosis and treatment had improved patient outcomes. However, the survival gains are stage-specific^2^. Liver resection, transplantation, ablation, or transarterial chemoembolization benefits patients in early or intermediate stages^3^, while treatment for late-stage HCC has remained challenging. The standard first-line systemic drug against advanced HCC is sorafenib (SF, BAY 43-9006, Nexavar), which is currently known as a ferroptosis inducer^4^, exerting only limited effects on overall survival and time to tumor progression^5,6^. Therefore, it is imperative to explore novel and effective therapeutic target to improve the cellular sensitivity to SF.

In recent years, ferroptosis has been confirmed to be an essential mechanism in SF treatment^7^. It is a kind of finely controlled cell death featured with iron-dependent accumulation of lipid hydroperoxides^8^. When HCC cells were exposed to SF, the cystine/glutamate antiporter system (system x_c_^−^) was targeted. Subsequently, the cystine uptake was blocked and the biosynthesis of glutathione (GSH) was inhibited, resulting in accumulation of lipid peroxidation products, and eventually inducing ferroptosis. Meanwhile, SF also induces autophagic flux in cells. When autophagy flux is initially induced, MTORC1 dissociates from the ULK1/2/ATG13 complex, leading to dephosphorylation of the complex^9^. Then, phagophore nucleation occurs, recruiting the ATG14/BECN1/PIK3C3/PIK3R4 complex^10^. Next, two ubiquitin-like conjugation systems are involved in phagophore elongation^11^. When de novo autophagosome formation is complete, they are delivered to the lysosome, initiating lysosomal degradation.

Actually, autophagy is a targetable pathway regulating cellular sensitivity to ferroptosis^12^. The crosstalk between autophagy and ferroptosis has attracted more and more attentions in the recent years. Autophagy inhibition by bafilomycin A1 or chloroquine, or lack of key autophagy associated gene (for example: ATG5, ATG7), will partially suppressed ferroptotic events through ferritinophagy inactivation, reducing the turnover of iron-binding ferritin and the iron release^13–15^.

CircRNAs primarily originate from back-splicing events of pre-mRNAs. The covalently bonded loops are highly stable and abundant^16^. The deregulation of circRNAs is closely related to various human diseases, including cancer^17–19^. In HCC, several studies have revealed that circRNAs are involved in multiple malignant behaviors^20^. However, the functions and mechanisms of circRNAs in SF treatment are still not comprehensively understood. In the current research, we found a novel circRNA derived from the *IARS* gene (ID from circBase: hsa_circ_0008367), named *cIARS*. *cIARS* was proven to be a promoter of ferroptosis in HCC cells after SF treatment, which was partially attributed to the activation of autophagy and ferritinophagy. The underlying molecular mechanism of *cIARS-*mediated ferroptosis was also clarified in this study.

## Results

### Circular transcript cIARS (hsa_circ_0008367) is significantly upregulated in SF-treated HCC cells

**Fig. 1:**
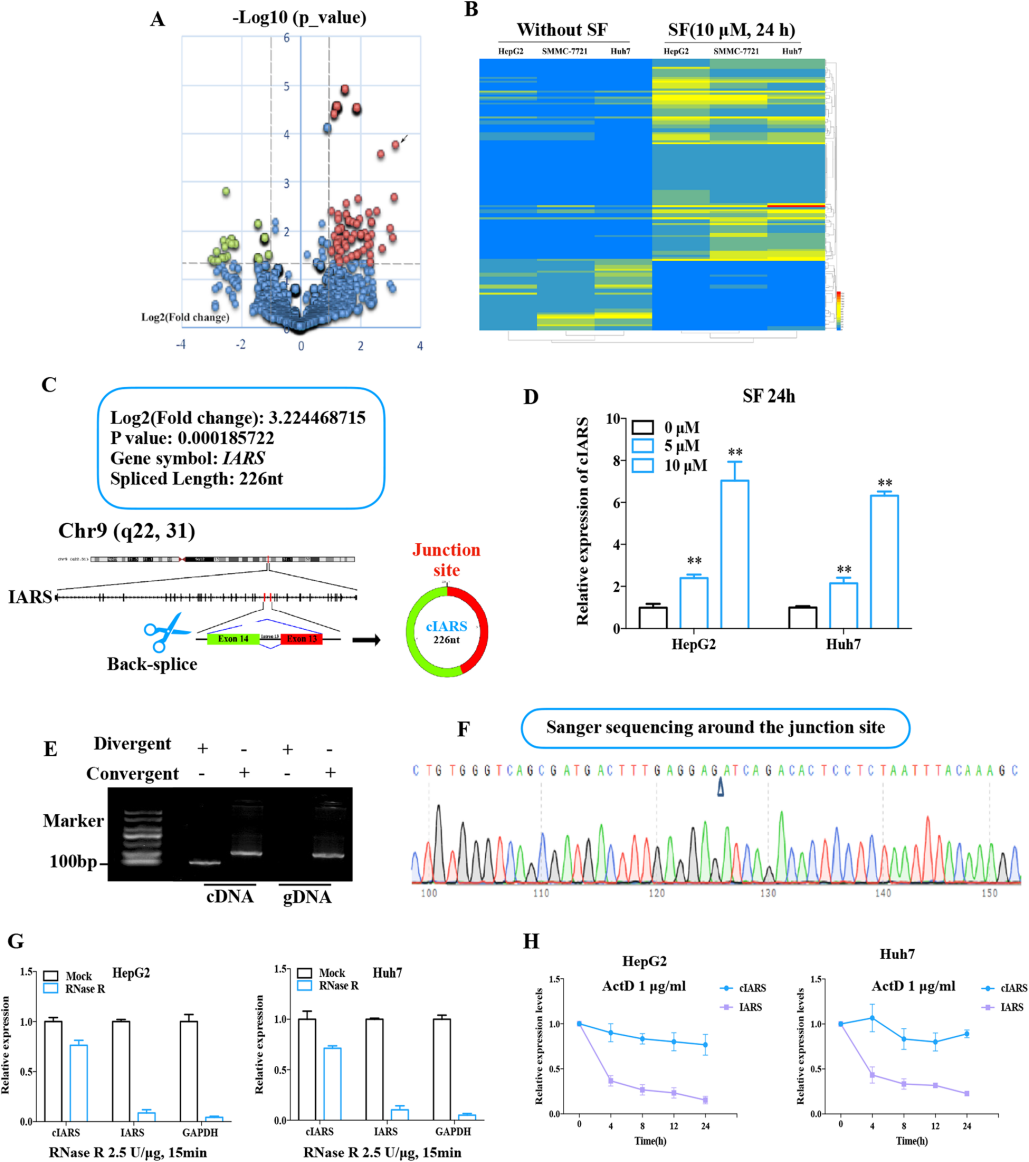
Circular transcript *cIARS* (hsa_circ_0008367) is significantly upregulated in SF-treated HCC cells. RNA-seq was performed in three pairs of SF-treated (10 μM, 24 h) and untreated HCC cell lines (HepG2, SMMC-7721 and Huh7). **a** The differentially expressed circRNAs were visualized with volcano plots; the −log10 (*p*-value) and the log2 (fold change) are plotted on the *y* and *x* axes, respectively. The dashed lines signify the filtering criteria (*p* < 0.05, fold change ≥ 2). Upregulated circRNAs are shown in red, and downregulated circRNAs are shown in green. The most highly expressed circRNA was marked by a black arrow. **b** Hierarchical cluster analysis showing the 137 differentially expressed circRNAs. The six columns represent the six different cellular samples. Each row indicates a circular transcript, and the colors represent the abundance of the transcripts. **c** Schematic diagram of the back-splicing transcript generated from linear *IARS*. Exon 13 and exon 14 are colored red and green respectively. **d** Relative expression of *cIARS* after SF treatment for 24 h in the HCC cell lines. **e** Divergent and convergent primers for *cIARS* were applied to amplify both cDNA and gDNA. Agarose gel electrophoresis visualized the products. **f** The sequence around the junction site was confirmed through Sanger sequencing. The empty triangle indicates the junction site. **g** RNase R exoribonuclease (2.5 U/μg, 37 °C, 15 min) was applied to remove the linear transcripts from cellular extracts, leaving circRNAs behind. qPCR was applied to assess the resistance of RNAs to RNase R. **h** RNA decay assay evaluating the stability of *cIARS* and *IARS* by qPCR after ActD (1 μg/ml) administration. The error bars represent the standard deviation (SD) of at least three independent experiments. **p < 0.01.

RNA-seq was performed in three pairs of SF-treated and untreated HCC cell lines (HepG2, SMMC-7721 and Huh7) to screen differentially expressed circRNAs. Cells were treated with 10 μM of SF for 24 h. 11367 unique circRNAs were found. Among these circRNAs, 3963 (35%) had already been reported in circBase^21^, and the other 7404 (65%) circRNAs were newly discovered. Among the 3963 annotated circRNAs, 2506 (63%) were of extremely low abundance with fragments per kilobase million (FPKM) values <1 in cellular samples and were thus excluded from this study. The other 1457 circRNAs comprised two groups: 1320 presented no significant changes in expression levels after SF treatment, and the other 137 were differentially expressed circRNAs (fold change ≥ 2, *p*-value < 0.05). The volcano plots demonstrated that there were 102 upregulated and 35 downregulated transcripts (Fig. 1a). The results of hierarchical clustering are displayed in a heatmap (Fig. 1b) generated with Heatmap Illustrator^22^ and suggest that there were two different clusters of transcripts. The most highly expressed circRNA was *cIARS* (hsa_circ_0008367) and was marked with black arrow in the volcano plots. According to circBase, *cIARS* is an exonic circRNA ~226 nt in length originating from the exon 13 and exon 14 of the *IARS* gene on chr9: 95030455–95032265 (Fig. 1c). qPCR showed that the relative expression levels of *cIARS* were significantly higher in SF-treated cell lines than in untreated ones (Fig. 1d). Both convergent and divergent primers of *cIARS* were applied for amplification. The band of *cIARS* was only observed in cDNA sample not the genomic DNA (Fig. 1e). Sanger sequencing further validated that the sequence around the junction site (about 100 bp around the site) was consistent with the result of RNA-seq and CircInteractome database^23^ (Fig. 1f). In addition, *cIARS* was much more resistant to RNase R (2.5 U/μg) (which degrades linear, but not circular, transcripts) than *IARS* and *GAPDH* (Fig. 1g). When actinomycin D (ActD, a transcription inhibitor) was added to HCC cells for the indicated time periods, *cIARS* was much more stable than its linear counterpart (Fig. 1h). These evidences suggested *cIARS* to be a highly abundant and stable circular transcript in HCC cells.

### cIARS is found to be a significant regulator of SF-induced ferroptosis

**Fig. 2:**
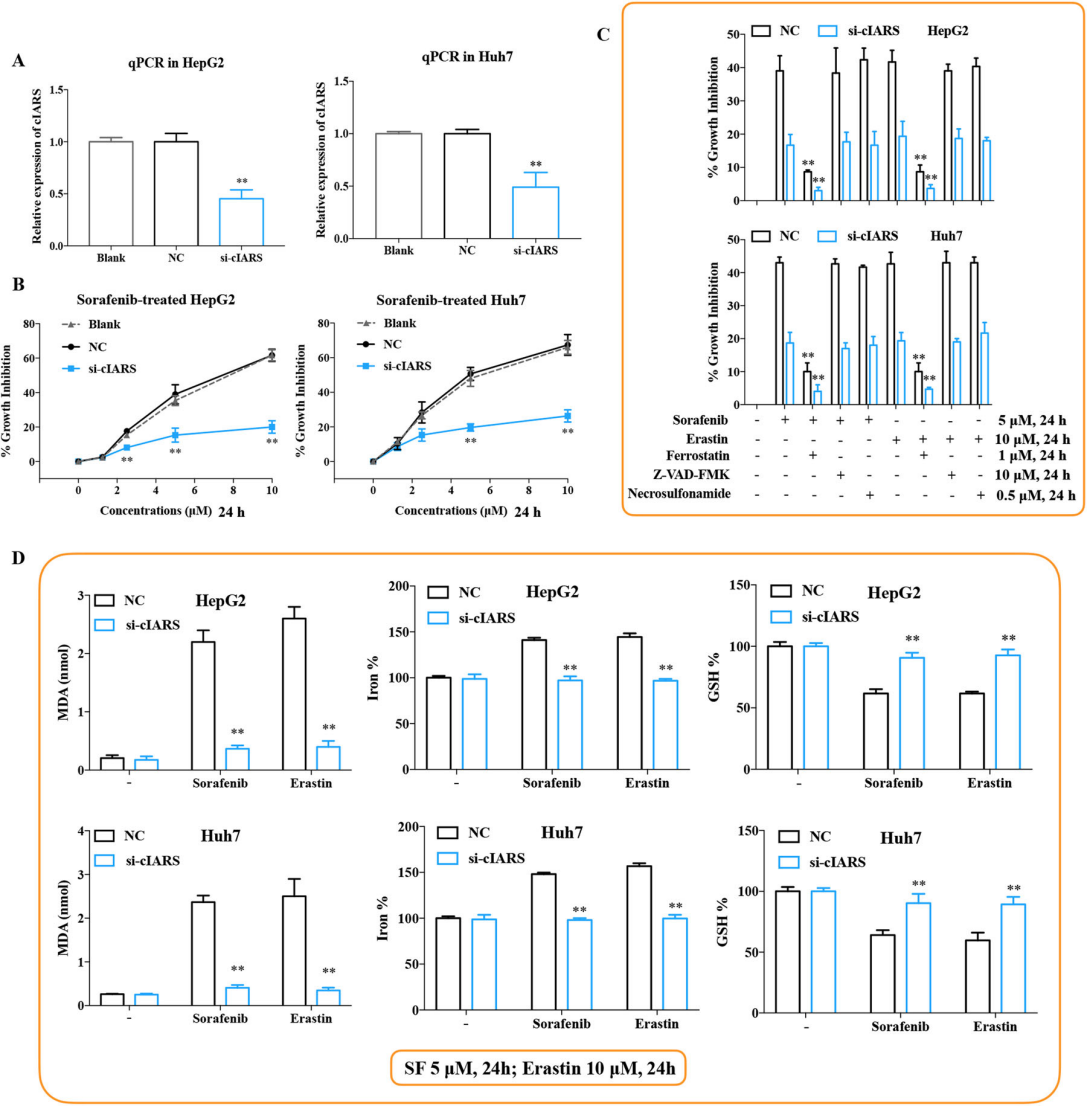
*cIARS* is found to be a significant regulator of SF-induced ferroptosis. **a** The expression levels of *cIARS* after transfection of si-cIARS or NC. **b** The evaluation of growth inhibition induced by SF in si-cIARS or NC transfected cells at the indicated concentrations for 24 h. **c** The evaluation of cytotoxicity of SF (5 μM, 24 h) and Erastin (10 μM, 24 h), with or without several inhibitors of cell death, including ferrostatin-1 (1 μM), ZVAD‐FMK (10 μM), or necrosulfonamide (0.5 μM). **d** The assessment of MDA, Fe^2+^, and GSH during SF treatment (5 μM, 24 h). **p < 0.01.

To clarify the biological role of *cIARS*, we first knockdown *cIARS* expression with a junction site-specific siRNA vector (si-cIARS). The effects of the si-cIARS was shown in Fig. 2a. CCK-8 assay showed that SF-induced growth inhibition was evidently weakened in si-cIARS transfected cells; Erastin-induced growth inhibition was also attenuated by si-cIARS (Fig. 2b, c). To determine the underlying mechanism, si-cIARS-introduced HCC cells were treated with various cell death inhibitors. Ferrostatin-1^24^, a specific ferroptosis inhibitor, significantly undermined the therapeutic effects of either SF or Erastin in both si-cIARS and NC transfected cells. However, ZVAD-FMK (an apoptosis inhibitor) and Necrosulfonamide (a necroptosis inhibitor) exerted no significant influence on SF or Erastin-induced growth inhibition (Fig. 2c). Simultaneously, malondialdehyde (MDA) and the level of Fe^2+^ were significantly reduced, while intracellular GSH obviously increased in the *cIARS* silencing cells following SF or Erastin administration (Fig. 2d). These evidences suggested *cIARS* to be a positive regulator of ferroptosis in HCC cells.

### cIARS positively regulates SF-induced autophagy and ferritinophagy

**Fig. 3:**
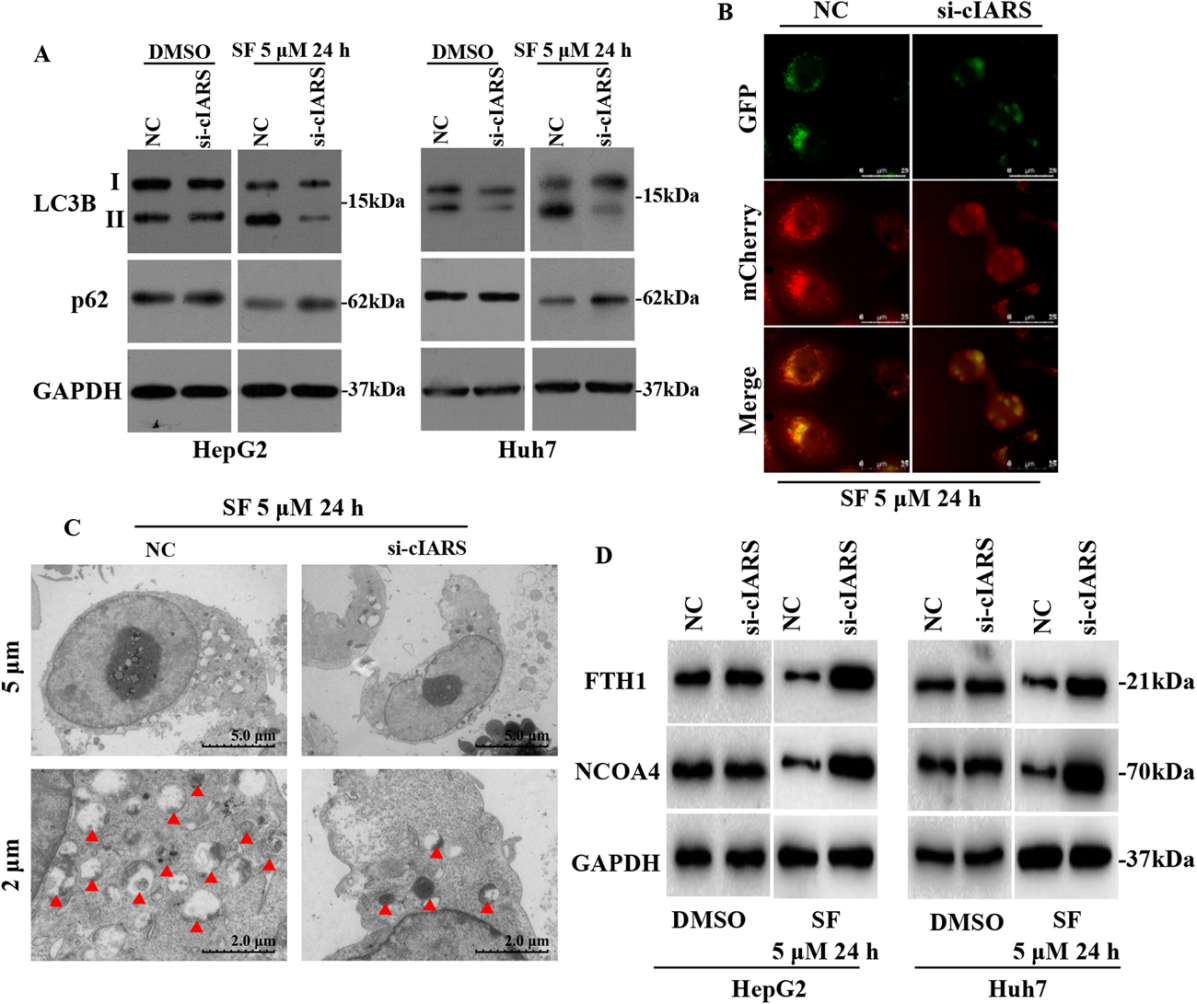
*cIARS* positively regulates SF-induced autophagy and ferritinophagy. **a** The expression levels of LC3B and p62 in si-cIARS-introduced cells with or without SF treatment. **b** Microscopic observation of autophagy flux after transfection of Ad-mCherry-GFP-LC3 for 24 h. mCherry-GFP-LC3 was visualized by fluorescence microscopy. The green fluorescent protein is acid sensitive and will be quenched in the acidic condition of lysosome, while the mCherry is stable at low pH. Thus, the red puncta represent autolysosomes and the yellow puncta, generated from the merge of both green and red signals, represent autophagosomes. The autophagosomal and autolysosomal abundance was measured by the number of puncta: lower density of yellow and red puncta suggested lower level of autophagic flux. **c** Visualization of autophagic compartments via TEM. The red arrowheads indicate double-membraned vacuoles. **d** The assessment of the protein levels of FTH1 and NCOA4 in si-cIARS or NC transfected cells with or without SF treatment (5 μM, 24 h).

*cIARS* was also found to be an autophagy regulator. Western blot (WB) assay showed that *cIARS* knockdown significantly decreased LC3 lipidation and increased p62 accumulation (Fig. 3a). Either autophagosomes or autolysosomes were observed via microscopic examination after Ad-mCherry-GFP-LC3 adenovirus transfection. This experiment is applied for concurrent observation of autophagosome and autolysosome. The signal of green fluorescent protein will be quenched during fusion of autophagosome and lysosome. Thus, the red signal of mCherry indicates autolysosome and the merge of green and red signals (yellow puncta) indicates autophagosome. si-cIARS significantly decreased the amount of red (autolysosome) and yellow (autophagosome) puncta per cell, demonstrating an inhibition of autophagy flux (Fig. 3b). TEM visually suggested the autophagic compartments. si-cIARS decreased the number of double-membraned vacuoles to a relatively low level (Fig. 3c). These results showed that *cIARS* is a positive autophagy regulator in SF-treated HCC cells. Furthermore, the protein levels of FTH1 and NCOA4, the substrate and cargo receptor of ferritinophagy, were determined by WB assay. si-cIARS resulted in remarkable accumulation of both FTH1 and NCOA4 (Fig. 3d). This finding indicated that the ferroptotic events in SF-treated HCC cells, positively regulated by *cIARS*, may be partially associated with autophagy and ferritinophagy.

### cIARS specifically interacted with RBP ALKBH5 (AlkB Homolog 5, RNA demethylase)

**Fig. 4:**
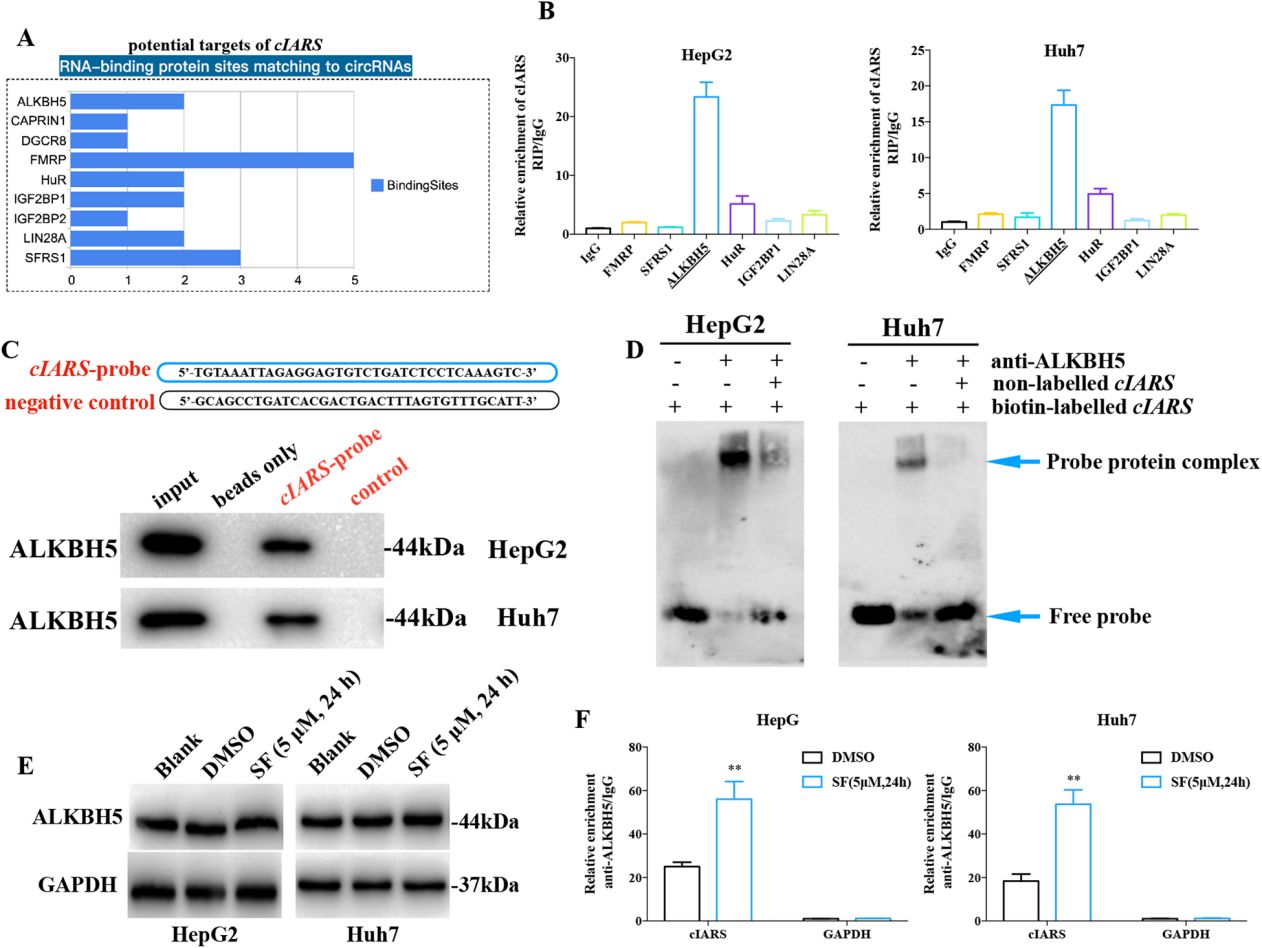
*cIARS* specifically interacts with ALKBH5. **a** Prediction analysis of the interaction between *cIARS* and RBPs through CircInteractome. **b** RIP assay evaluating the physical interaction between *cIARS* and the six candidate RBPs in HCC cells. **c**, **d** RNA EMSA and RNA pulldown evaluating the physical interaction between *cIARS* and ALKBH5. **e** The protein levels of ALKBH5 in HCC cells in the absence or presence of SF treatment. **f** The assessment of the physical binding of *cIARS* and ALKBH5 by RIP assay, in the absence or presence of SF treatment. **p < 0.01.

Recent studies have revealed that circRNA–RBP interaction has important roles in diverse biological processes. According to CircInteractome database, we found six RBPs bearing at least two binding sites matching to *cIARS*, including FMRP (Fragile X Mental Retardation 1), SFRS1 (Serine And Arginine Rich Splicing Factor 1), ALKBH5, HuR (ELAV like RNA binding protein 1), IGF2BP1 (Insulin Like Growth Factor 2 MRNA Binding Protein 1), and LIN28A (Lin-28 Homolog A) (Fig. 4a). RBP immunoprecipitation (RIP) assay in both HepG2 and Huh7 cell lines demonstrated that the relative levels of *cIARS* in ALKBH5 enriched samples were much higher than in the other five RBPs (Fig. 4b). RNA pulldown and RNA EMSA were performed to determine the interaction using an anti-sense probe spanning the junction site of *cIARS*. The presence of specific bands demonstrated the physical binding of *cIARS* and ALKBH5 (Fig. 4c, d). Interestingly, SF administration had no influence on the protein levels of ALKBH5 in both HepG2 and Huh7 cells (Fig. 4e), but remarkably increased the *cIARS*–ALKBH5 interaction (Fig. 4f), which may be due to SF-induced expression of *cIARS*.

### cIARS repressed the role of ALKBH5 in the regulation of autophagy

**Fig. 5:**
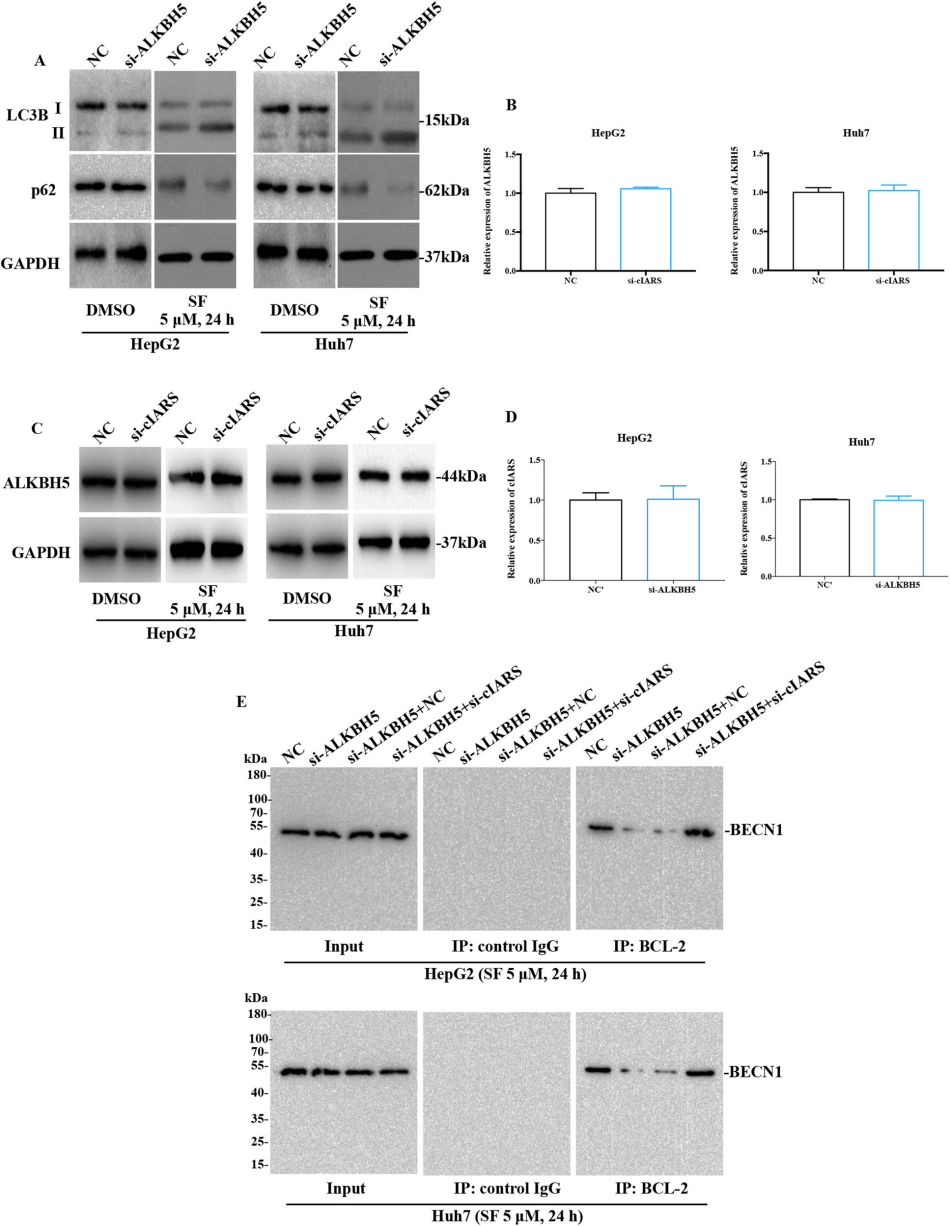
*cIARS* repressed the role of ALKBH5 in the regulation of autophagy. **a** The expression levels of LC3B and p62 in si-ALKBH5 or NC′ introduced cells with or without SF administration. **b**, **c** The relative expression of ALKBH5 mRNA and protein in si-cIARS or NC introduced HCC cells. **d** The relative expression of *cIARS* in si-ALKBH5 or NC′ transfected cells. **e** The assessment of the role of si-cIARS in ALKBH5-mediated interaction between BECN1 and BCL-2.

ALKBH5 had been previously proven to be an autophagy inhibitor in cancer^25,26^. However, its role in SF-treated HCC cells remains unclear. WB assay showed that siRNA against ALKBH5 (si-ALKBH5) significantly promoted the transformation of LC3B I to II and degraded p62 (Fig. 5a). This result suggested ALKBH5 to be a negative regulator of autophagy in HCC cells. qPCR and WB showed that si-cIARS failed to influence ALKBH5 mRNA and protein levels (Fig. 5b, c); similarly, si-ALKBH5 also had no impact on the relative expression of *cIARS* (Fig. 5d). A previous study revealed that si-ALKBH5 promoted the dissociation of BECN1 and BCL-2^26^, a key step during phagophore nucleation. In SF-treated HCC cells, we gained similar results via IP assay. More importantly, this process can be effectively blocked by *cIARS* silencing (Fig. 5e). These results demonstrated that *cIARS* repressed the biological role of ALKBH5 in autophagy.

### cIARS regulates ferroptosis through ALKBH5-mediated autophagy

**Fig. 6:**
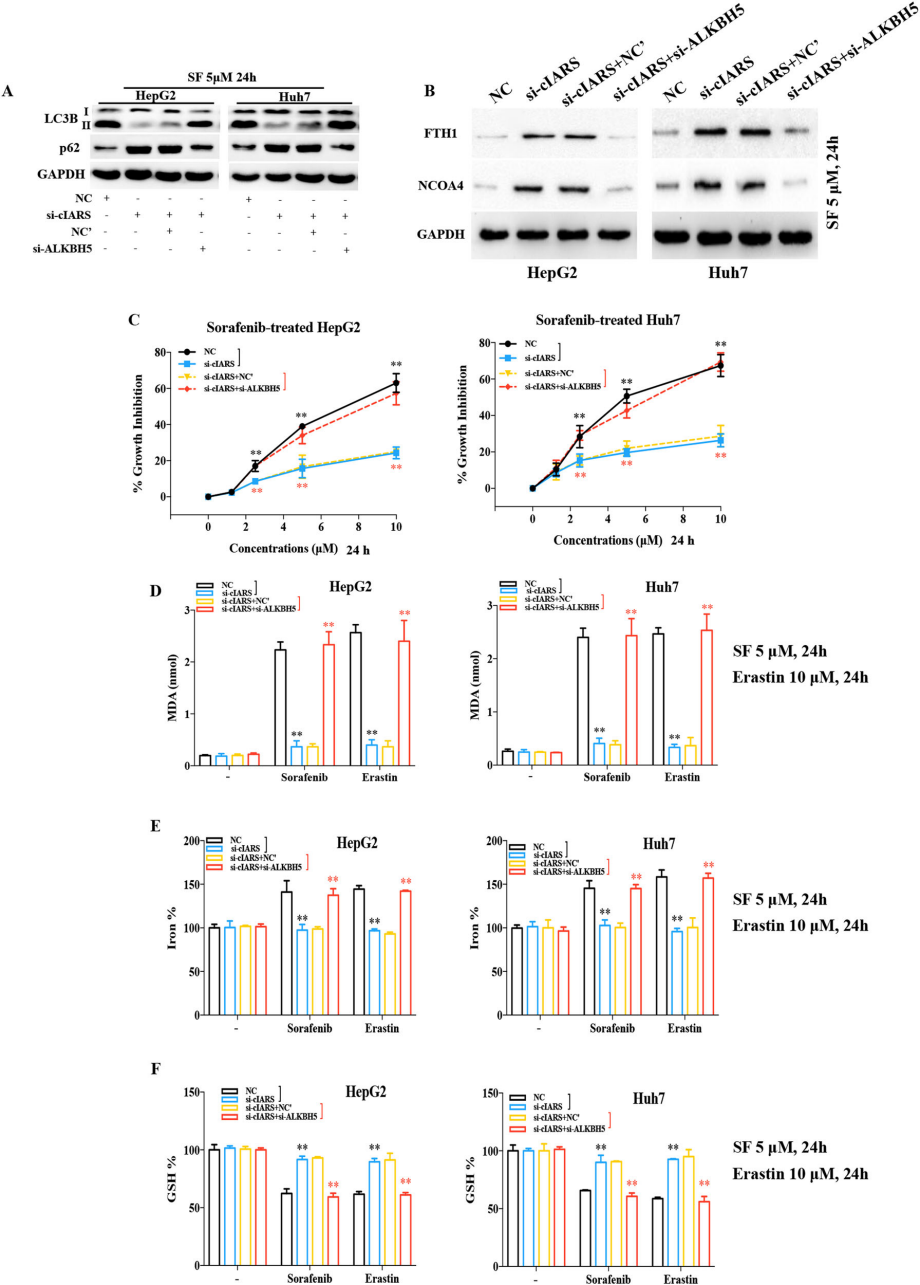
*cIARS* regulates ferroptosis through ALKBH5. **a**, **b** The evaluation of the role of si-ALKBH5 in si-cIARS-regulated autophagy and ferritinophagy. The WB assay demonstrated that: the effect of si-cIARS on LC3 lipidation and p62 accumulation can be significantly reversed by si-ALKBH5 in SF (5 μM, 24 h) treated HCC cells (**a**); the effect of si-cIARS on the accumulation of FTH1 and NCOA4 can be evidently rescued by si-ALKBH5 in either HepG2 or Huh7 cells treated by SF (5 μM, 24 h) (**b**). **c** Identification of the role of ALKBH5 in *cIARS*-regulated growth inhibition by CCK-8. The results demonstrated that: si-ALKBH5 significantly reversed si-cIARS-mediated growth inhibition in SF administered HCC cells. **d**–**f** The rescue experiments evaluating the effects of si-ALKBH5 in the si-cIARS-mediated ferroptotic events. The relative levels of MDA and iron were obviously decreased and the level of GSH increased in si-cIARS introduced HCC cells; these impacts can be effectively reversed by ALKBH5 knockdown. The black whiskers indicated the difference between the “si-cIARS” and “NC” groups; while the red ones indicated the difference between the “(si-cIARS+NC’)” and “(si-cIARS+si-ALKBH5)” groups. **p < 0.01.

To explore whether *cIARS*-regulated ferroptosis via ALKBH5, we performed several phenotype rescue experiments. First, si-ALKBH5 successfully rescued the effects of si-cIARS in autophagic flux and ferritinophagy (Fig. 6a, b). Second, ALKBH5 knockdown effectively re-intensified the SF cytotoxicity, which was remarkably impaired by si-cIARS (Fig. 6c). Third, si-cIARS-mediated decrease of MDA, Fe^2+^ and increase of GSH, can be rescued by si-ALKBH5 (Fig. 6d–f).

Taken together, a novel circRNA in HCC was revealed in our research. We partially clarified its role and mechanism in ferroptosis.

## Discussion

As a novel class of noncoding RNA, circular transcripts have attracted widespread attention. However, circRNA-regulated ferroptosis in human diseases has not been widely investigated. A small portion of studies focused on the circRNA-mediated autophagy. For instance, Chen et al.^27^ reported that *circHIPK3* depletion significantly induced autophagy via *miR-124-3p*-STAT3-PRKAA/AMPKa axis and there was an antagonistic regulation on autophagy between *circHIPK3* and linear *HIPK3*. Du WW et al.^28^ showed that the oncogenic *circDnmt1*-stimulated autophagy flux in breast carcinoma via interaction with both p53 and AUF1. These findings demonstrated the potential of circRNAs in autophagy regulation and prompted us to explore the role of circRNA in ferroptosis, which had been identified as an autophagic cell death^12^.

In our research, we delineated a mechanism of circRNA-mediated ferroptosis during SF treatment in HCC cells. circRNA *cIARS* (hsa_circ_0008367) was screened from RNA-seq analysis. Phenotypically, *cIARS* positively regulated ferroptosis, which may be partially dependent on autophagy and ferritinophagy. Mechanistically, *cIARS* physically interacted with RBP ALKBH5 and negatively regulated its role in autophagy.

To comprehensively investigate the complicated circRNA network, it is essential to deeply evaluate its binding partners. In this study, *cIARS* is found to be an interactor of RBP ALKBH5, which had previously been reported to be a N6-methyladenosine (m^6^A) eraser. Its role in autophagy regulation is completely different in different tissues and diseases^25,26,29–31^. In lung cancer^25^, ALKBH5 upregulation stabilized UBE2C, an autophagy inhibitor, with maintenance of lower m^6^A level. In ovarian cancer^26^, ALKBH5 inhibited autophagy through activating PI3K-Akt-mTOR signaling pathway, stabilized BCL-2 mRNA and promoted the interaction between BCL-2 and BECN1. Herein, *cIARS* was proven to be a pivotal regulator of autophagy, ferroptosis, and ferritinophagy, depending on negatively regulating the biological role of ALKBH5, an autophagy inhibitor in HCC cells.

In this study, the *cIARS*–ALKBH5 axis was demonstrated to be a key mechanism regulating ferroptosis during SF treatment. Further studies are still needed at the clinical and mechanistic levels.

## Materials and methods

### Cell culture and transfection assay

Under humidified conditions with 5% CO_2_ at 37 °C, the HCC cell lines HepG2, SMMC-7721, and Huh7 (bought from the National Infrastructure of Cell Line Resource) were cultured in Dulbecco’s Modified Eagle’s medium (HyClone, Logan, UT, USA) with 10% fetal bovine serum (Gibco, Carlsbad, CA, USA) and penicillin (100 U/ml)-streptomycin (100 µg/ml) solution in the medical research center of Shandong Provincial Qianfoshan Hospital, Shandong First Medical University. All of the cell lines mentioned in this research were cultured within ten passages. The transfection experiments were performed, aided by Lipofectamine 3000 (Life Technologies, Carlsbad, CA, USA). The RNA oligonucleotides in this work were designed and constructed by GenePharma (Shanghai, China), including siRNAs against *cIARS* or *ALKBH5*, and the corresponding negative controls (NC and NC’). Sequences were shown as follows (5′–3′): si-cIARS: GAC UUU GAG GAG AUC AGA CAC; si-ALKBH5: GGA UAU GCU GCU GAU GAA ATT; NC/NC′: UUC UCC GAA CGU GUC ACG U.

### Cell Counting Kit-8 (CCK-8) assay

The growth inhibition rate of HepG2 and Huh7 cells was assessed by the CCK-8 (Dojindo Laboratories, Japan) assay. The blank or transfected cells (5 × 10^3^ cells per well) were seeded into 96-well plates with three replicate wells. After the treatment with SF or Erastin or various cell death inhibitors (Ferrostatin-1, ZVAD-FMK or Necrosulfonamide) for 24 h, a 10 µl volume CCK-8 reagent was added to each well. Then, measuring the absorbance at 450 nm after incubation with the CCK-8 solution at 37 °C for 2 h.

### RNA extraction and analysis

Expression profiles of genome-wide circRNAs in three pairs of HCC cell lines (before and after SF treatment) were explored on an Illumina HiSeq 4000 platform (by Novogene, Beijing, China). The cDNA from divergent primers was subjected to Sanger sequencing (by Zhonghong Boyuan Biological Technology, Jiangxi, China). Agarose gel electrophoresis was used to detect the qPCR amplification of cDNA and genomic DNA after applying with the divergent and convergent primers of *cIARS*.The relative fold changes in expression were calculated with the formula 2^−ΔΔCt^. The sequences of all the primers were listed as follows (5′–3′): *cIARS* F: AGC GAT GAC TTT GAG GAG ATC A, R: CCC AGT AGC ACA GGT CAT TG; *IARS* F: CAT ATC CAG TTT CTC CAT CGG A, R: TGG ATT TTC CAG GAG CAA TAC T; *ALKBH5* F: GCA AGG TGA AGA GCG GCA TCC, R: GTC CAC CGT GTG CTC GTT GTA C; *U6* F: CTC GCT TCG GCA GCA CAT A, R: ATT TGC GTG TCA TCC TTG CG; *GAPDH* F: CAG AAC ATC ATC CCT GCC TCT AC, R: ATG AAG TCA GAG GAG ACC ACC TG.

### Ribonuclease R (RNase R) assay

RNase R assay (R0301, 20 U/µl, Geneseed, Guangzhou, China) was used for the identification of circRNA. According to the manufacturer’s guidance: 2 µl 10× Reaction Buffer and 2.5 U RNase R/µg RNA were mixed to the total RNA, and then added RNase-Free Water to form a 20 µl reaction solution system. After digestion with RNase R for 15 min at 37 °C, the enzyme then was inactivated at 70 °C for 10 min and then directly perform reverse transcription reaction. The qPCR assay was used to determine the relative expression of cIARS, IARS and GAPDH compared to the mock group.

### Actinomycin D (ActD) assay

ActD assay (HY-17559, MedChem Express, New Jersey, USA) was used to detect the stability of RNA. 1 μg/ml ActD reagent was used to treat HepG2 and Huh7 cells. After 0, 4, 8, 12, or 24 h of administration, the total RNA was extracted respectively to determine the relative expression of cIARS or IARS by qPCR assay.

### Western blot analysis

Radioimmunoprecipitation assay buffer (RIPA) was applied to lyse cells. Total proteins were then harvested and quantified with bicinchoninic acid assays (Beyotime, Shanghai, China). The target proteins were separated through 10% SDS-PAGE and transferred to polyvinylidene fluoride membranes (Merck Millipore, Burlington, MA, USA). The membranes were blocked with nonfat milk, incubated with primary antibodies, and then incubated with secondary antibody diluted at a ratio of 1:10,000 (Jackson ImmunoResearch, West Grove, PA, USA). The primary antibodies were anti-LC3B (2775, Cell Signaling Technology, Beverly, MA, USA), anti-p62 (88588, Cell Signaling Technology), anti-HuR (ab28660, Abcam, Cambridge, MA, USA), anti-SFRS1 (ab133689), anti-FMRP (ab17722), anti-ALKBH5 (ab195377), anti-IGF2BP1 (ab82968), anti-LIN28A (ab46020), anti-FTH1 (ab65080), anti-NCOA4 (ab86707), anti-BCL-2 (ab32124), anti-BECN1 (ab62557), and anti-GAPDH (ab9485). The protein signals were visualized with enhanced chemiluminescence detection reagents (ECL, Millipore, Burlington, MA, USA) and quantified with Image Lab software (Bio-Rad, Hercules, CA, USA).

### Observation of autophagy flux

Ad-mCherry-GFP-LC3 adenovirus (Servicebio Technology, Wuhan, China) was transfected into HCC cells. After 24 h incubation, cells were observed and photographed using a fluorescence microscope (Olympus FSX100, Tokyo, Japan). Autophagic compartments were finely observed through transmission electron microscopy (TEM, HT7700, HITACHI, Tokyo, Japan). The identification of autophagic vacuoles in TEM images was mainly based on the 3rd edition of the Guidelines for the Interpretation of Assays for Monitoring Autophagy^32^.

### Immunoprecipitation

RIP and IP were performed to confirm the RNA–protein and protein–protein interactions using RIP Kit (17-700, Millipore) and Immunoprecipitation Kit (ab206996) according to the manufacturer’s guidance. RIP related details had been described in our previous studies^33,34^. The IP assay consists of four steps, including antibody binding, beads preparation, bead capture, and elution. The volume of the antibody binding system was made up to 500 µl with lysis buffer containing the protease inhibitor cocktail and gently mixed for 4 h; the protein A/G sepharose (30 µl/reaction) was washed twice with wash buffer, centrifuged at 2000 × *g* for 2 min and aspirated the supernatant between washes.

### RNA pulldown assay

Pierce™ Magnetic RNA-Protein Pull-Down Kit (Thermo) was applied to evaluate RNA–protein interaction using biotin-labeled junction-specific probe and its negative control (designed and synthesized by Viagene Biotech, Jiangsu, China). Detailed procedure strictly followed the manufacturer’s guide. *cIARS*-probe: 5′-TGT AAA TTA GAG GAG TGT CTG ATC TCC TCA AAG TC-3′; Negative control: 5′-GCA GCC TGA TCA CGA CTG ACT TTA GTG TTT GCA TT-3′.

### RNA EMSA

The LightShift Chemiluminescent RNA EMSA Kit (ThermoFisher Scientific) was applied to evaluate the interaction between *cIARS* and ALKBH5, according to the manufacturer’s guidance. The details were described in our as previous research^34^.

### Lipid peroxidation assay

The relative level of MDA was evaluated through Lipid Peroxidation Kit (ab118970) according to the manufacturer’s guidance.

### Iron assay

The relative level of intracellular iron was determined by an Iron Assay Kit (ab83366) according to the manufacturer’s instructions.

### Glutathione assay

The relative level of intracellular GSH was assessed through a GSH Colorimetric Detection Kit (CS0260, Sigma-Aldrich, St. Louis, USA) according to the manufacturer’s instructions.

### Statistical analysis

The statistical analyses in this work were carried out using Prism 7. The results from at least three independent tests are shown as the mean value ± standard deviation (SD). The mean values of two groups were compared via unpaired Student *t*-tests. “*p-*value < 0.05” was defined as significant. *p < 0.05, **p < 0.01.

## References

[1] Bray F (2018). Global cancer statistics 2018: GLOBOCAN estimates of incidence and mortality worldwide for 36 cancers in 185 countries. CA Cancer J. Clin..

[2] Siegel RL, Miller KD, Jemal A (2018). Cancer statistics, 2018. CA Cancer J. Clin..

[3] Forner A, Reig M, Bruix J (2018). Hepatocellular carcinoma. Lancet.

[4] Li J (2020). Ferroptosis: past, present and future. Cell Death Dis..

[5] Llovet JM (2008). Sorafenib in advanced hepatocellular carcinoma. N. Engl. J. Med..

[6] Cheng AL (2009). Efficacy and safety of sorafenib in patients in the Asia-Pacific region with advanced hepatocellular carcinoma: a phase III randomised, double-blind, placebo-controlled trial. Lancet Oncol..

[7] Dixon SJ (2014). Pharmacological inhibition of cystine-glutamate exchange induces endoplasmic reticulum stress and ferroptosis. Elife.

[8] Stockwell BR (2017). Ferroptosis: a regulated cell death nexus linking metabolism, redox biology, and disease. Cell.

[9] Jung CH (2009). ULK-Atg13-FIP200 complexes mediate mTOR signaling to the autophagy machinery. Mol. Biol. Cell.

[10] Itakura E, Mizushima N (2010). Characterization of autophagosome formation site by a hierarchical analysis of mammalian Atg proteins. Autophagy.

[11] Geng J, Klionsky DJ (2008). The Atg8 and Atg12 ubiquitin-like conjugation systems in macroautophagy. ‘Protein modifications: beyond the usual suspects’ review series. EMBO Rep..

[12] Zhou, B. et al. Ferroptosis is a type of autophagy-dependent cell death. *Semin. Cancer Biol*. 10.1016/j.semcancer.2019.03.002 (2019). 10.1016/j.semcancer.2019.03.002 30880243

[13] Torii S (2016). An essential role for functional lysosomes in ferroptosis of cancer cells. Biochem. J..

[14] Gao M (2016). Ferroptosis is an autophagic cell death process. Cell Res..

[15] Hou W (2016). Autophagy promotes ferroptosis by degradation of ferritin. Autophagy.

[16] Memczak S (2013). Circular RNAs are a large class of animal RNAs with regulatory potency. Nature.

[17] Bi W (2018). CircRNA circRNA_102171 promotes papillary thyroid cancer progression through modulating CTNNBIP1-dependent activation of beta-catenin pathway. J. Exp. Clin. Cancer Res..

[18] Su Y (2020). circRIP2 accelerates bladder cancer progression via miR-1305/Tgf-beta2/smad3 pathway. Mol. Cancer.

[19] Chen J (2017). Circular RNA profile identifies circPVT1 as a proliferative factor and prognostic marker in gastric cancer. Cancer Lett..

[20] Wang, M., Yu, F. & Li, P. Circular RNAs: characteristics, function and clinical significance in hepatocellular carcinoma. *Cancers* **10**, 10.3390/cancers10080258 (2018). 10.3390/cancers10080258 PMC6116001 30072625

[21] Glazar P, Papavasileiou P, Rajewsky N (2014). circBase: a database for circular RNAs. RNA.

[22] Deng W, Wang Y, Liu Z, Cheng H, Xue Y (2014). HemI: a toolkit for illustrating heatmaps. PLoS ONE.

[23] Dudekula DB (2016). CircInteractome: A web tool for exploring circular RNAs and their interacting proteins and microRNAs. RNA Biol..

[24] Dixon SJ (2012). Ferroptosis: an iron-dependent form of nonapoptotic cell death. Cell.

[25] Guo J (2018). Deregulation of UBE2C-mediated autophagy repression aggravates NSCLC progression. Oncogenesis.

[26] Zhu H (2019). ALKBH5 inhibited autophagy of epithelial ovarian cancer through miR-7 and BCL-2. J. Exp. Clin. Cancer Res..

[27] Chen X (2020). Circular RNA circHIPK3 modulates autophagy via MIR124-3p-STAT3-PRKAA/AMPKalpha signaling in STK11 mutant lung cancer. Autophagy.

[28] Du WW (2018). A circular RNA circ-DNMT1 enhances breast cancer progression by activating autophagy. Oncogene.

[29] Song H (2019). METTL3 and ALKBH5 oppositely regulate m(6)A modification of TFEB mRNA, which dictates the fate of hypoxia/reoxygenation-treated cardiomyocytes. Autophagy.

[30] Chen, Y. et al. m(6)A mRNA methylation regulates testosterone synthesis through modulating autophagy in Leydig cells. *Autophagy* 1–19, 10.1080/15548627.2020.1720431 (2020). 10.1080/15548627.2020.1720431 PMC8007139 31983283

[31] Li G (2020). Bone-derived mesenchymal stem cells alleviate compression-induced apoptosis of nucleus pulposus cells by N6 methyladenosine of autophagy. Cell Death Dis..

[32] Klionsky DJ (2016). Guidelines for the use and interpretation of assays for monitoring autophagy (3rd edition). Autophagy.

[33] Yang G (2019). circ-BIRC6, a circular RNA, promotes hepatocellular carcinoma progression by targeting the miR-3918/Bcl2 axis. Cell Cycle.

[34] Liu, B. et al. CircBACH1 (hsa_circ_0061395) promotes hepatocellular carcinoma growth by regulating p27 repression via HuR. *J. Cell Physiol*. 10.1002/jcp.29589 (2020). 10.1002/jcp.29589 32003018

